# Ultrasonic Pretreatment in Synthesis of Caprylic-Rich Structured Lipids by Lipase-Catalyzed Acidolysis of Corn Oil in Organic System and Its Physicochemical Properties

**DOI:** 10.3390/foods8110566

**Published:** 2019-11-11

**Authors:** Chonghui Yue, Hongyan Ben, Junwen Wang, Tiantian Li, Guoping Yu

**Affiliations:** College of Food Science, Northeast Agricultural University, Changjiang Road, Harbin 150036, China; ychad321@163.com (C.Y.); 15765513086@139.com (H.B.); 18846820334@163.com (J.W.); Tiantian19980511@163.com (T.L.)

**Keywords:** acidolysis, lipase, caprylic, corn oil, ultrasonic pretreatment, structured lipids

## Abstract

The current work was to evaluate the lipase-catalyzed acidolysis of corn oil with caprylic acid (CA) in organic system under bath ultrasonic pretreatment and to analyze the physicochemical properties of structured lipids (SLs). Under optimum conditions (Novozym 40086 lipase, 200 W ultrasound power, 10 min ultrasound pretreatment time, 12% dosage of lipase, Triacylglycerol (TAG)/Free fatty acids (FFA): 1/8, 40 °C for 6 h), a 45.55% CA incorporation was obtained (named SLs-U). The highest CA incorporation was 32.75% for conventional method at reaction time of 10 h (named SLs-N). The predominant TAG types of SLs were MLM (medium-, long- and medium-chain-type TAGs) and MLL (medium-, long- and long-chain-type TAGs). X-ray diffraction analysis revealed that both SLs-U and SLs-N present β form. Differential scanning calorimetry (DSC) analysis showed that both SLs-U and SLs-N show a lower melting and crystallization temperature than corn oil. This study suggested that bath ultrasonic pretreatment can accelerate lipase-catalyzed acidolysis synthesis of MLM structured lipids in an organic system, and two kinds of structured lipids show similar physicochemical properties.

## 1. Introduction

Cardiovascular disease, which caused by higher serum total cholesterol (TC) and low-density lipoprotein cholesterol (LDL-C) and lower high-density lipoprotein cholesterol (HDL-C), has becomea widespread epidemic disease in the past few years [1]. In recent years, the “low calorie lipids” have attracted much attention because the functionality and nutrition which can relieve cardiovascular disease has promoted the research on structured lipids (SLs) enriched with medium-chain fatty acids such as caprylic (C8:0) and capric (C10:0) acids. Structured lipid is defined as triacylglycerols that can be modified by chemical and enzymatic methods to change fatty acids composition and positional distribution.

Medium-chain fatty acids (MCFAs) have smaller molecular size and higher solubility which can be rapidly hydrolyzed in gastrointestinal tract and are directly transported to the liver via hepatic portal circulation [2,3,4]. Furthermore, medium-chain fatty acids can provide quick delivery of energy and rarely be stored in the body. Some studies showed that consumption of MLCTs (medium- and long-chain triacylglycerols) can promote macrophage reverse cholesterol transport and reduce total cholesterol and low-density lipoprotein [2]. However, medium-chain fatty acids cannot be used as the only source of dietary fat since they can cause gastrointestinal problems [5]. Therefore, there is an increasing interest in the SLs containing medium-chain fatty acids at sn-1,3 position and functional polyunsaturated fatty acid in the sn-2 position [6].

The enzymatic modification of triacylglycerols has attracted much attention in recent years because of the properties such as reaction specificity, milder reaction conditions, and ease product recovery [3,7,8]. Another advantage of enzymatic reactions is that they can provide also solvent-free reactions, avoiding also the use of corrosive acids [9,10]. Also, the enzymatic method is widely used in the synthesis of novel and high-added value compounds, such as wax esters, that contribute significant merit to the production of novel structured lipids [11,12]. More specifically, the development of biorefineries can result to high-added value compounds production, which then can be used as raw materials for the formation of structured lipids [11,12,13]. Currently, using the medium-chain fatty acids as acyl donors and vegetable, animal, fish, or microbial oilas the source of glycerol backbone and long-chain fatty acids through lipase-catalyzeda cidolysis reaction to produce MLM (medium-, long- and medium-chain-type TAGs) structured lipids has become a most common method. Corn oil is an appropriate substrate for the synthesis of MLM because of its high linoleic acid (50–60%) which is beneficial for human health, mainly at the sn-2 position in the TAGs (Triglycerides) [14,15]. Previous study investigated the synthesis of structured lipid by lipase-catalyzed acidolysis of corn oil with conjugated linoleic acid [16]. Nevertheless, synthesis of MLM structured lipids which use corn oil as glycerol backbone has been rarely investigated.

Ultrasound irradiation has been widely used because of its cavitational collapse effect which causes the reduction in the mass transfer limitations that accelerates lipase-catalyzed reactions [17,18]. Also, the ultrasound irradiation can increase the interface area of lipase and substrate and reduce particle size. Furthermore, the ultrasound irradiation can change the active site of the lipase and result in an increase or decrease in enzyme activity. Currently, the ultrasonic technologies mainly include ultrasonic microtip probe and ultrasonic bath. The ultrasonic microtip probe can transfer the energy directly and ultrasonic bath transfer the energy indirectly. However, the long-time ultrasonic treatment consumes significant amounts of energy, particularly the ultrasonic microtip probe. Therefore, the ultrasonic pretreatment iswidely used at the beginning of reaction for saving energy, improving the biocatalyst performance [19], accelerating the reaction [20], and increasing the product yields [18]. The application of ultrasonic bath in many investigations was often used for biodiesel production and diacylglycerol production, which can accelerate the reaction efficiency [21,22]. Currently, the efficiency of lipase-catalyzed acidolysis and the incorporation ratio of MCFAs are relatively low [4,5,6]. Also, there is rarely investigation on the ultrasound bath irradiation pretreatment for lipase-catalyzed acidolysis of structured lipids in a solvent system.

The aim of this paper was to investigate the effect of ultrasonic pretreatment using an ultrasound bath in the production of SLs enriched with MCFA at sn-1 and 3 position by lipase-catalyzed acidolysis of corn oil with caprylic acid. The effects of different reaction conditions, including ultrasonic pretreatment time, ultrasonic power, enzyme dosage, substrate molar ration, reaction temperature, and reaction time on the incorporation of caprylic acid were investigated. The physicochemical properties of MLM structured lipids produced with ultrasonic pretreatment compared to SLs produced without ultrasonic pretreatment were analyzed.

## 2. Materials and Methods

### 2.1. Materials

Corn oil “Chang shouhua” was purchased from a local supermarket (Harbin, China). Three commercialimmobilized lipases named Novozym 40086 (*Aspergillus oryzae*), Lipozyme TL IM (*Thermomyces lanuginosus*) and Novozym 435 (*Candida antarctica*) were obtained from Novozymes (Beijing, China). Caprylic acid (C8:0, 99%) was purchased from Sigma-Aldrich Chemical (Shanghai, China). Pancreatic lipase was purchased from Sigma-Aldrich Chemical Co., Ltd. (Shanghai, China). Silicic acid GF 254 TLC plates (10 × 20 cm) were purchased from Qingdao Haiyang (Qingdao, China). A mixture of standards consisting of 37 fatty acid methyl esters was obtained from Supelco (Shanghai, China). 

### 2.2. Lipase-Catalyzed Acidolysis with Ultrasonic Pretreatment

Indirect ultrasonic pretreatment assisted acidolysis reaction was carried out using an ultrasonic water bath (KQ-400KDE, Kunshan, China, power rating of 400 W and frequency of irradiation of 40 kHz). The reaction power could be adjusted from 160 W to 400 W. 

The ultrasonic pretreatment was performed before the acidolysis reaction. The corn oil (1 mmol) was mixed with CA at the substrate molar ratio of 1:8 in a 50 mL screw-capped centrifuge tube, and the lipase (12% by total weight of reactants) and 5 mL *n*-hexane were added. The tube was first incubated in the ultrasonic water bath and partly emerged into the water for ultrasonic pretreatment at designed pretreatment conditions. After ultrasonic pretreatment, the mixture was transferred to an orbital shaking water bath at 50 °C for 8 h at 200 rpm. The zero-time (t0) of the reaction is the time after ultrasonic pretreatment reaction and before transferring to orbital shaking water bath. Different operating conditions studied in the present work are the different ultrasonic power (160, 200, 240, 280, 320, 360, and 400 W), different pretreatment time (5, 10, 20, 30, and 40 min), different enzyme loading (3%, 6%, 9%, 12%, 15%, and 18%,W/W), different substrate molar ratio (1:2, 1:4, 1:6, 1:8, and 1:10), different reaction temperature (30, 40, 50, 60, and 70 °C) and different reaction time (2, 4, 6, 8, 10, and 12 h). The MLM structured lipids after lipase-catalyzed acidolysis with ultrasonic pretreatment (named SLs-U) and conventional method without ultrasonic pretreatment (named SLs-N) were used for future analysis.

### 2.3. Removal of Free Fatty Acids (FFA)

The lipases were removed by filtration when the reaction was finished. 0.5 M KOH hydroalcoholic solution (30% ethanol) was added into the reaction product to neutralize the extra free fatty acids. The mixture was shaken violently and stratified statically. The hexane phase and hydroalcoholic phase were decanted. The upper layer (hexane layer) was collected and filtered to remove water. The upper layer was dried by using anhydrous sodium sulfate. The solvent was evaporated using a rotary evaporator at 37 °C. The samples were stored at −20 °C.

### 2.4. Triacylglycerol Isolation by TLC

The reaction productions were isolated using thin-layer chromatography (TLC) plates with hexane/diethyl ether/acetic acid (70:30:1.5, *v*/*v*/*v*), and detected with 0.2% 2,7-dichlorofluorescein in methanol solution under UV light. The triglyceride bands were scraped off the TLC plate and extracted using diethyl ether [23]. The isolated TAGs were used to analyze total FA composition and sn-2 position.

### 2.5. Fatty Acids Composition Analysis

The fatty acids composition was analyzed as fatty acid methyl esters (FAME) as described by P.A. Nunes et al. (2011) [24] with some modifications. FAME were identified and quantified using a GC-14B gas chromatograph (Shimadzu, Tokyo, Japan), equipped with a flame ionization detector (FID), and a DB-WAX capillary column (30 m × 0.25 mm × 0.25 μm). The detector and injector temperatures were set at 250 °C. The oven temperature program was as follows: 100 °C for 4 min, temperature increase to 180 °C at 15 °C/min, a plateau at 180 °C for 4 min, temperature increase to 230 °C at 7.5 °C/min, and a final plateau at 230 °C for 15 min. The FAMEs were identified by comparing their retention times of the peaks with the respective standards of FAMEs, and the FAME contents were obtained by area normalization and expressed as mass percentage.

### 2.6. Pancreatic Lipase Catalyzed sn-2 Position

The positional distribution of the fatty acid in modified TAG was determined by pancreatic hydrolysis [25]. In brief, 1 mL of Tris-HCl buffer (1 M, pH7.6), 0.5 mL of sodium cholate solution (0.05%, *w*/*v*), 0.2 mL CaCl_2_ (2.2%, *w*/*v*), and 10 mg of pancreatic lipase were added to samples. The mixture was incubated at 40 °C for 5min, vortexed vigorously. Then, 1 mL of hydrochloric acid (6 mol/L), 1 mL diethyl ether, and 1 mL ethanol were added and the mixture was vortexed and centrifuged at 2180 g for 5 min. Diethyl ether was dried and evaporated under nitrogen. The sample was developed with hexane/diethyl ether/acetic acid (50/50/1, *v*/*v*/*v*) by using TLC plates. The 2-MAG band was scraped and extracted with diethyl ether. The extraction was methylated and analyzed by GC.

### 2.7. TAG Composition Analysis by Ultra-HPLC

The TAG composition was analyzed by ultra-HPLC as described by Jiyuan Lu et al. with some modifications [26]. The samples were analyzed by Waters w2695 HPLC system equipped with an ODS-2 HYPERSIL C18 column (5 μm, 4.6 × 250 mm). A mixture of acetonitrile and Isopropyl alcohol (40:60, *v*/*v*) used as mobile phase with an initial flow rate of 0.1 mL/min, increased to 0.18 mL/min after 5 min and maintained this rate to the end. Column temperature was set as 45 °C. The equivalent carbon number (ECN) was used to predict the elution order (ECN = CN−2DB), where CN is the total carbon number without glycerol and DB is the total number of double bonds in the FAs.

### 2.8. FTIR Analysis

The Fourier-transform infrared (FTIR) was determined using a Shimadzu 8400S Fourier transform IR spectrophotometer (FTIR) (Kyoto, Japan). The FTIR spectra were recorded in the range of 600–4000 cm^−1^ at a resolution of 4 cm^−1^ by 32 scans [27]. 

### 2.9. DSC Analysis

The crystallization and melting profiles of modified TAGs were measured by a differential scanning calorimeter fitted with a liquid nitrogen cooling system according to the previous method described by Heet al. (2016) with slight modifications [28]. Accurately weighted 10 mg samples and placed into a standard aluminium crucible and crimped in place. A weight empty aluminium pan was set as a control. The program was as follow: the sample was heated from 20 °C to 80 °C at 20 °C/min and maintains this temperature for 10 min to destroy any crystal memory. The sample was immediately cooled from 80 °C to −40 °C at rate of 5 °C/min to obtain the crystallization curve. Then the sample was maintained at −40 °C for 10 min and heated to 80 °C at a rate of 5 °C.

### 2.10. Statistical Analysis

All acidolysis reactions were carried out in triplicate. The obtained results were presented as the mean ± standard deviations. The results were analyzed by one-way analysis of variance (ANOVA) using statistical software (version 5.1, CoStat, Monterey, CA, USA). The results were considered statistically for *p* values < 0.05.

## 3. Results and Discussion

### 3.1. Screening of Commercial Immobilized Lipases

**Figure 1:**
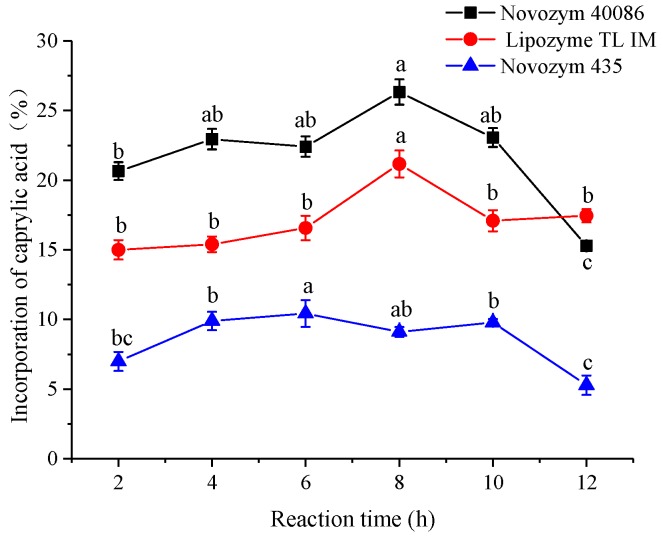
Effect of reaction time on incorporation of caprylic acid into corn oil by different lipases. Reaction conditions: 200 W ultrasonic power, 10 min pretreatment time, 1:6 corn oil/CA molar ratio, 12% enzyme dosage, and 5 mL of hexane, 50 °C. Different letters indicate significant differences.

As shown in Figure 1, three commercial immobilized lipases from different sources were screened for their reaction efficiency to incorporate caprylic into corn oil after different reaction times in an organic solvent system. Figure 1 indicated three lipases presented the highest incorporate ratio around 8 h. The incorporate ratio decreased after 8 h. The Novozym 40086 lipase presented the highest incorporate ratio compared with Lipozyme TL IM and Novozym 435. The CA incorporate ratio was as follows, in descending order: Novozym 40086 (24.88%) > Lipozyme TL IM (21.17%) > Novozym 435 (10.42%). The Novozym 435 lipase showed the lowest ratio of CA incorporation. When compared with Novozym 40086, Lipozyme TL IM is a lipase that suit for the synthesis of short chain alkyl esters. Novozym 435 is a high thermostability lipase that commonly used in transesterification reaction [29]. Novozym 40086 is a lipase that has a maximum enzyme activity at 40–70 °C. In this study, the range of reaction temperature was 40–70 °C. Furthermore, the ultrasound irradiation can make the enzyme structure become flexible, and the enzyme may shift into its active configuration. The different lipases have different stereostructure, and they can deserve different effects under the same treatment conditions of ultrasonication [30]. Therefore, the Novozym 40086 was the optimal lipase to analyze the effect of ultrasound pretreatment on CA incorporation ration in the experiments.

### 3.2. Effect of Ultrasonic Pretreatment Parameters on the Acidolysis Reaction

**Figure 2:**
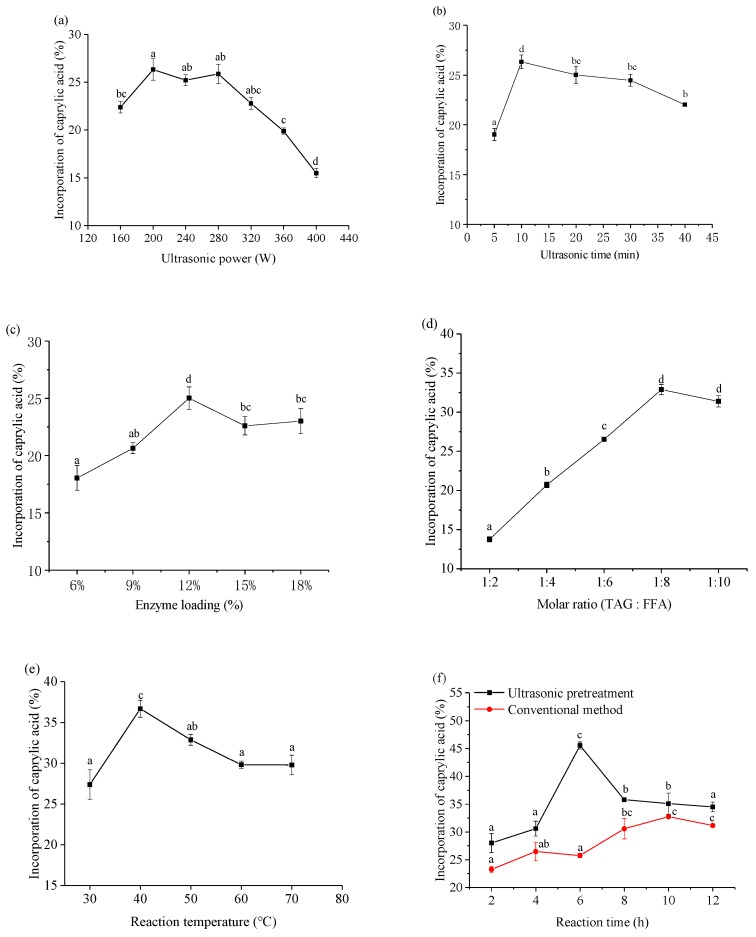
Optimization of reaction conditions for MLM (medium-, long- and medium-chain-type TAGs) structured lipids synthesis by enzymatic acidolysis in organic system assisted by bath ultrasonic pretreatment. Reaction conditions: (**a**), 10 min pretreatment time, 1:6 corn oil/CA molar ratio, 12% enzyme dosage, and 5 mL of hexane, 50 °C; (**b**), 200 W ultrasonic power, 200 W ultrasonic power, 1:6 corn oil/CA molar ratio, 12% enzyme dosage, and 5 mL of hexane, 50 °C;(**c**), 200 W ultrasonic power, 10 min pretreatment time, 12% enzyme dosage and 5 mL of hexane, 50 °C;(**d**), 200 W ultrasonic power, 10 min pretreatment time, 12% enzyme dosage and 5 mL of hexane, 50 °C;(**e**), 200 W ultrasonic power, 10 min pretreatment time, 1:8 corn oil/CA molar ratio, 12% enzyme dosage, and 5 mL of hexane; (**f**), 200 W ultrasonic power, 10 min pretreatment time, 1:8 corn oil/CA molar ratio, 12% enzyme dosage, and 5 mL of hexane, 40 °C. Different letters indicate significant differences.

The ultrasonic power is a critical factor affecting the incorporation ratio of CA. The effect of ultrasonic power (160, 200, 240, 280, 320, 360, 400 W) was investigated to obtain the maximum CA incorporation ratio with a 10 min pretreatment time, 1:6 corn oil/CA molar ratio, 12% enzyme dosage, 5 mL of hexane, and temperature of 50 °C (Figure 2a). The CA incorporation significantly increased with increasing ultrasonic power from 160 W to 200 W and decreased with increasing ultrasonic power from 280 W to 400 W. There was no obvious difference in CA incorporation between 200 W and 280 W. The ultrasound can cause mechanical vibration of the liquid when ultrasound passes through a liquid medium. The mechanical vibration leads to an alternative vibration and rarefaction action. During this alternative action, occurrence or formation of bubbles or cavities subsequently develop and further implodes [31]. Ultrasound energy helps reduce mass-transfer limitation and enhances the interfacial area [32]. But, the higher ultrasound power during the pretreatment resulted in enzyme inactivation. Therefore, 200 W of ultrasonic power was used for further experiments.

Many studies reported that ultrasound treatment time has significant effects on reaction rate and enzyme activity [33,34]. However, it is much more feasible and efficient when the ultrasound pretreatment irradiation is used as a method before lipase-catalyzed reaction in industrial production. The effects of ultrasonic pretreatment time of 5, 10, 20, 30 and 40 min on the acidolysis reaction are shown in Figure 2b. When the ultrasound pretreatment time increased from 5 to 10 min, the CA incorporation rapidly increased. However, the CA incorporation significantly decreased when the ultrasonic pretreatment time exceed 10 min. Some studies revealed that ultrasonic could reduce mass transfer limitations by reducing the particle size and increasing the contact area of enzyme and substrate. The phenomenon was obvious when using lipase to catalyze reactions in organic solvent. However, long time for high density ultrasonic treatment might produce lots of heat thus induce enzyme inactivity. Furthermore, the structure of lipase was changed under long-time ultrasonic treatment. Shah et al. (2008) reported that ultrasonic irradiation can enhanced lipase activity in organic system, and the circular dichroism (CD) spectra showed that ultrasonic irradiation could change the lipase tertiary structure slightly [35]. Considering the CA incorporation, energy saving, and reaction efficiency, 10 min was selected as the optimum ultrasonic pretreatment time. 

The enzyme dosage was varied from 6% to 18% according to the weight of the substrates, with the mole ratios of the reactants held constant. As shown in Figure 2c, the incorporation of CA was increased rapidly with the increase in enzyme dosage from 6% to 12%. The percentage of CA incorporation ratio increased from 18.05% to 25.01%. This result was in agreement with the Öztürk Tarık et al. (2010), who found that the maximum ratio of CA incorporation (25.1%) into fish oil was achieved with an enzyme load of 12% [15]. The results may be due to the available of extra enzyme active sites for catalytic reaction, and consequently increase the rate of acidolysis reaction, thus resulting in the increased incorporation of acyl donors [36]. However, at higher enzyme dosage (12–15%), the CA incorporation was decreased. However, no obvious increase in the CA incorporation was found when the enzyme loading was increased beyond 15%. This result was probably due to the enzyme aggregation which inhibits substrate diffusion, causing the reaction rate saturation. Considering the cost of enzyme and CA incorporation, 12% was selected as the ideal dosage of Novozym 40086.

As shown in Figure 2d, a significant increase of CA incorporation was observed once the substrate molar ratio increased from 1:2 to 1:8. The CA incorporation was increased from 13.75% to 32.89%. However, by further increasing the substrate molar ratio did not result in an increase in CA incorporation. This was probably due to the reduced ability of enzyme active sites to accommodate more substrates in the presence of excess substrate. On the other hand, this result was probably ascribed to the inhibition of Novozym 40086 at excessive FFAs (free fatty acids) by acidifying the micro aqueous phase surrounding the lipase. High levels of FAs produce more free or ionized carboxylic acid groups, leading to adsorption of water from the interface. Similar result was reported by Abed et al. (2018), who investigated the influence of molar ratio on the acidolysis of microbial oil with CA catalyzed by Lipozyme RM IM [6]. Considering the cost of materials as well as the content of CA, a substrate molar ratio of 1:8 was selected for the following investigation.

The temperature plays an important role in lipase-catalyzed acidolysis reactions due to the improvement of diffusion efficiency and lipase activation or lipase inactivation [37]. In this study, the temperature was set at 30, 40, 50, 60, 70 °C to examine the effect of reaction temperature on the incorporation of CA, and the results are shown in Figure 2e. As shown in Figure 2e, the CA incorporation was rapidly increased with the increasing of reaction temperature from 30 °C to 40 °C. The CA incorporation was increased from 27.39% to 36.71%. However, CA incorporation was decreased when reaction temperature beyond the 40 °C, indicating the deactivation ofthe enzyme. The results were in agreement with Kim et al. (2010), who reported the synthesis of structured lipids by the acidolysis of borage oil with caprylic acid using lipases [38]. The result was probably ascribed to the decrease of viscosity of substrate and the increase of effective collision of reactants, thus promote the reaction. The increase of temperature also affects the bubble formation and collapse caused by ultrasound, a higher temperature has a negative effect on ultrasound effect. And the higher temperature causes the oxidation of the polyunsaturated fatty acids [39]. According to the results, 40 °C was selected as the optimum reaction temperature for subsequent experiments.

The effect of reaction time on the incorporation of CA was studied under ultrasonic pretreatment method and conventional method (without ultrasonic pretreatment). As shown in Figure 2f, CA incorporation after ultrasonic pretreatment was increased with increasing reaction time from 2 h to 6 h. However, the CA incorporation was decreased when the reaction time exceeds 6 h. This result was probably ascribed to the inactivation of lipase. Under conventional method, the CA incorporation was increased with increasing reaction time from 2 h to 10 h, and a slowly decrease was observed when the reaction time exceeded 10 h. As shown in Figure 2f, the CA incorporation using ultrasonic pretreatment was consistently higher than that of the conventional method over the10 h of reaction. The reaction achieved a higher efficiency in a relatively short reaction time (6 h) under ultrasonic pretreatment and yielded a 45.55% CA incorporation. However, the optimum reaction time for conventional method was 10 h and yielded a 32.75% CA incorporation. Many researches on lipase-catalyzed acidolysis of triacylglycerols with caprylic for producing MLM structured lipids shown that the CA incorporation is between 20% and 30% [5,6]. Based on our data, the efficiency of synthesis of structured lipids was significantly improved under the ultrasonic pretreatment condition. This obvious difference between the ultrasound pretreatment and conventional method may be due to the cavitation efficiency from the ultrasonic pretreatment. The results suggested that indirect ultrasonic pretreatment could be applied to promote the acidolysis for synthesis of structured lipids enriched with medium-chain fatty acids. The 6 h reaction time for ultrasonic pretreatment method and 10 h reaction time for conventional method were used in further experiments.

### 3.3. Fatty Acid Composition and Positional Distribution

The fatty acids compositions of corn oil, SLs-U, and SLs-N are shown in Table 1. As shown in Table 1, the major fatty acids in corn oil were linoleic (C18:2, 47.66%), followed by oleic (C18:1, 25.60%), palmitic (C16:0, 11.72%), and stearic (C18:0, 1.53%) acids. The sn-1 and sn-3 positions were predominantly occupied by linoleic (40.82%), oleic (26.32%) and palmitic (15.46%) acids. After lipase-catalyzed acidolysis with or without ultrasonic pretreatment, the contents of caprylic (C8:0) were obviously increased. However, the FA content of SLs-U and SLs-N had obvious difference, which means that the lipase-catalyzed acidolysis with ultrasonic pretreatment had significant effect on FA compositions in samples. The contents of caprylic acid of SLs-U and SLs-N were 45.55% and 32.75%, respectively. On the other hand, at sn-1,3 positions of SLs-U and SLs-N, the caprylic and linoleic acid were found to be the predominant fatty acids. After lipase-catalyzed acidolysis, there was no significant change in sn-2 linoleic acid of SLs-U and SLs-N. However, the SLs-U and SLs-N showed a significant increase in the content of oleic acid at sn-2 position from 24.16% to 31.76% and 33.88%, respectively. The SLs containing medium-chain fatty acids at sn-1,3 position and functional polyunsaturated fatty acid in the sn-2 position would have desirable physicochemical and nutritional properties, such as lower melting point and low caloric value [24]. The structured lipids synthesized by ultrasonic pretreatment would present important medical and functional properties which would be used as a low caloric lipids and functional lipids in the future.

### 3.4. TAG Composition Analysis

**Figure 3:**
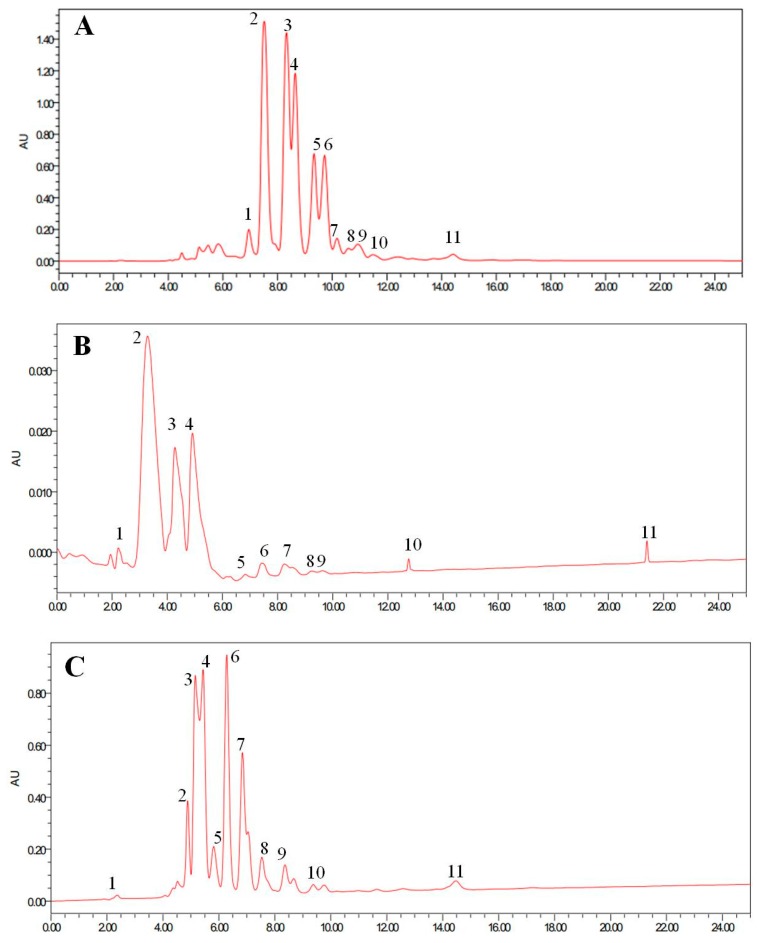
HPLC chromatograms of corn oil (**A**), SLs-U (**B**), and SLs-N (**C**).

The different types of TAG in corn oil, SLs-U, and SLs-N were separated by HPLC and the results are shown in Figure 3. As shown in Figure 3A, the major TAG types of corn oil were identified as LLL (L: Long Chain Fatty Acids) (peak 2, 3, 4, 5, 6 with ECN, C44, C46, C48). Compared with corn oil, the TAG types of SLs-U (Figure 3B) and SLs-N (Figure 3C) were predominantly by MLM and MLL. As shown in Figure 3B, the final TAG compositions for SLs-U were 58.4% MLM (peak 1, 2, 3, 4 with ECN, C28, C30, C32, C34) and 31.1% MLL (peak 4, 5 6 with ECN, C36, C38, C40). Meanwhile, as shown in Figure 3C, the final composition for SLs-N were 35.2% MLM (peak 2, 3, 4, 5 with ECN, C28, C30, C32, C34) and 25.1% MLL (peak 6, 7, 8 with ECN, C36, C38, C40). The synthesized SLs showed an obvious difference in the TAG types. The results were in agreement with Wang Yingyao et al. (2012), who studied the lipase-catalyzed acidolysis of canola oil with caprylic acid to produce MLM structured lipids (SLs) [23]. The results in this study also showed that the ultrasonic pretreatment method presents a positive effect on the synthesis of MLM structural lipids.

### 3.5. FTIR Spectra and X-Ray Spectra Analysis

**Figure 4:**
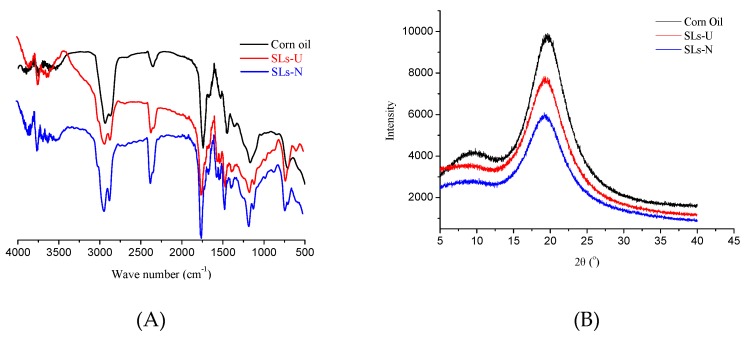
FTIR spectra (**A**) and X-ray spectra (**B**) of corn oil, SLs-U, and SLs-N.

The FTIR spectra and X-ray spectra for corn oil, SLs-U and SLs-N are shown in Figure 4. As shown in Figure 4A, the major absorption bands of SLs-U, SLs-N, and corn oil were similar, with some differences in the individual absorption bands. The three types of lipids showed the same broad stretching peak near 2924 cm^−1^ and 1465 cm^−1^ represent the -C-H (CH_2_) asymmetrical stretching, the brand near 1450 cm^−1^ represent -C-H (CH_3_) in plane flexural vibration and the bands at 754 cm^−1^ and 701 cm^−1^ represented -(CH_2_)n of the swing vibration. The bands at 1300 cm^−1^ and 1000 cm^−1^ represented the -C-O stretching and -HC= CH-bending [27]. The differences between two types of SLs and corn oil were observed at 1101 cm^−1^ which SLs had the absorption peaks at 1101 cm^−1^ and the corn oil did not have the same absorption peaks. The results could attribute to the increase of medium-chain FA which is saturated fatty acids and the reduction of unsaturated fatty acids. 

The polymorphism of lipid could be analyzed by the short spacing of the crystal. Three representative polymorphism forms in lipids were α, β′, and β form. According to the reports by Chai et al. (2016), the short spacings were at 4.12 Å for *α* form, 4.2 and 3.8 Å for β′ form, and 4.53 Å for β form [40]. As shown in Figure 4B, corn oil showed a high-intensity diffraction peak at 2θ of approximately 19.1° corresponded to the β form, with a short spacing of 4.6 Å. Thesimilar result was reported by Zhang Zhen et al. (2015) [41]. Also, SLs-U and SLs-N showed a high-intensity diffraction peak at 2 θ of approximately 19.1º corresponded to the β form which was similar with corn oil. However, compared with corn oil, the diffraction peak at 2 θ approximately 19.1° of SLs-U and SLs-N became weaker. The intensity of diffraction peak at 2 θ of approximately 19.1° was followed by cornoil > SLs-U > SLs-N. These results indicated that the amounts of β form in SLs-U and SLs-N were decreased after lipase-catalyzed acidolysis. A similar result was reported by Zhu Tingwei et al. (2018), who found that the β crystal was decreasing after lipase-catalyzed interesterified blend of palm stearin and vegetable oil [42]. 

### 3.6. Crystallization and Melting Profiles by DSC

**Figure 5:**
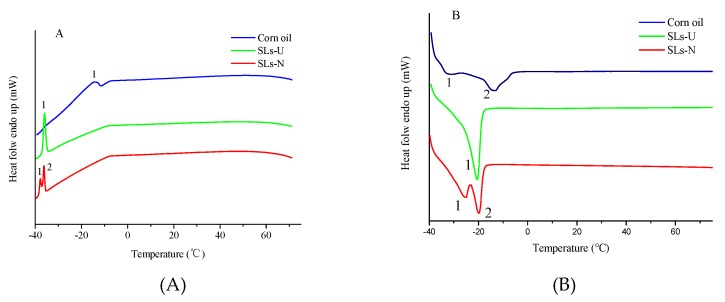
Differential scanning calorimetry (DSC) crystallization curves and melting curves ofcorn oil, SLs-U, and SLs-N (**A**, crystallization curves; **B**, melting curves).

Thermal profiles illustrated that the transition of heats and temperatures in the process of the melting and crystallization of lipids and provided a supplementary explanation for lipid composition. The crystallization and melting profiles of corn oil, SLs-U, and SLs-N are presented in Figure 5A,B, respectively. The chemical composition of lipids has an effect on their melting and crystallization profiles. The melting profiles are crucial for investigating physical form of oil and lipid in the human body. The corn oil showed a crystallization range from −14.24 °C and −7.96 °C with a peak at −11.60 °C (Figure 5A) and a melting range from −32.82 °C to −3.58 °C with two peaks (peak 1 and 2) at −32.82 °C and −13.11 °C (Figure 5B). SLs-U showed a crystallization range from −39.34 °C to −34.01 °C with a peak at −36.03 °C (Figure 5A) and a melting range from −24.93 °C to −17.68 °C with a peak at −20.42 °C (Figure 5B). SLs-N showed a crystallization range from–39.73 °C to −35.22 °C with two peaks (peak 1 and 2) at −37.86 °C and −36.21 °C. Also, for SLs-N, the melting curve appears two peaks (peak 1 and 2) at −25.25 °C and −19.71 °C in the range from −28.85 °C and −16.30 °C (Figure 5B). The melting and crystallization temperatures of SLs-U and SLs-N were lower than corn oil which indicating the structured lipid enriched with caprylic appeared to possess enhanced physicochemical properties over the native corn oil. The results were attributed to the incorporation of caprylic acid which having fewer carbon atoms. Lipids display a lower melting temperature when containing a high content of medium-chain fatty acids than those containing long-chain fatty acids [43]. The difference of crystallization and melting properties between SLs-U and SLs-N was probably due to the differences in the contents of medium-chain fatty acids and long-chain unsaturated fatty acids. The results were in according with reports by Abed et al. (2018), who illustrated that the SLs enriched with CA showed an obvious decrease in the melting and crystallization profiles in comparison with those of the native microbial oil [6].

## 4. Conclusions

In the present study, MLM structured lipids enriched with caprylic (MCFA) via lipase-catalyzed acidolysis of corn oil with or without ultrasonic pretreatment was investigated. The ultrasonic pretreatment method significantly improved the efficiency of MLM structured lipidssynthesis and shortened the acidolysis reaction time. Optimal reaction conditions for the pretreatment were 200 W ultrasonic power, 10 min pretreatment time, 12% dosage of Novozym 40086 lipase, TAG/FFA molar ratio of 1:8, reaction temperature 40 °C, and reaction time 6 h. The difference of fatty acids composition and TAG types between SLs-U and SLs-N was significant. No significant differences were observed between SLs-U and SLs-N in FTIR spectra and crystal form, while the thermal properties of SLs-U and SLs-N differed somewhat from corn oil. The difference between the physicochemical properties of structured lipids enriched with caprylic and native corn oil can probably due to the differences in their chemical compositions and structures. These results indicated that ultrasound pretreatment wasan efficient method to enhance lipase-catalyzed synthesis of MLM structured lipids in organic system, and couldbe used as an environmental and energy-saving method in industrial production.In addition, the ultrasound pretreatment method may be used to promote the enzymatic synthesis of novel and high-added value compounds that contribute significant merit to the production of novel food formulations, such as other kinds of structured lipids.

## Figures and Tables

**Table 1:** Fatty acids composition (%) and positional distributions of corn oil, SLs-U, and SLs-N.

Fatty Acids	Corn oil			SLs-U			SLs-N		
	Total	Sn-2	Sn-1,3	Total	Sn-2	Sn-1,3	Total	Sn-2	Sn-1,3
C8:0	ND	ND	ND	45.55 ± 0.65 ^a^	7.01 ± 0.02 ^a^	64.82 ± 0.43 ^a^	32.75 ± 0.29 ^b^	6.36 ± 0.03 ^b^	45.95 ± 0.78 ^b^
C16:0	11.72 ± 0.21 ^a^	4.24 ± 0.05 ^b^	15.46 ± 0.08 ^a^	5.31 ± 0.1 ^c^	4.42 ± 0.05 ^a^	5.76 ± 0.2 ^c^	6.44 ± 0.2 ^b^	4.43 ± 0.1 ^a^	7.45 ± 0.6 ^b^
C18:0	1.53 ± 0.2 ^a^	0.47 ± 0.04 ^a^	2.08 ± 0.2 ^a^	0.62 ± 0.1 ^c^	0.42 ± 0.03 ^b^	0.72 ± 0.6 ^c^	0.84 ± 0.5 ^b^	0.46 ± 0.2 ^a^	1.03 ± 0.6 ^b^
C18:1	25.60 ± 0.6 ^a^	24.16 ± 0.7 ^b^	26.32 ± 0.8 ^a^	13.23 ± 0.12 ^c^	31.76 ± 2.18 ^a^	3.97 ± 0.3 ^c^	16.54 ± 1.02 ^b^	33.88 ± 2.64 ^a^	7.87 ± 0.3 ^b^
C18:2	47.66 ± 0.8 ^a^	61.35 ± 0.8 ^a^	40.82 ± 1.42 ^a^	25.32 ± 1.14 ^c^	51.64 ± 2.06 ^b^	12.16 ± 1.2 ^c^	32.16 ± 0.8 ^b^	59.07 ± 0.7 ^a^	18.71 ± 0.5 ^b^
C18:3	0.51 ± 0.04 ^a^	0.73 ± 0.04 ^a^	0.40 ± 0.03 ^a^	0.33 ± 0.03 ^c^	0.35 ± 0.01 ^c^	0.32 ± 0.02 ^b^	0.42 ± 0.02 ^b^	0.51 ± 0.03 ^b^	0.37 ± 0.02 ^a^
C20:0	0.48 ± 0.03 ^a^	0.64 ± 0.04 ^a^	0.40 ± 0.03 ^a^	0.39 ± 0.01 ^c^	0.61 ± 0.01 ^b^	0.28 ± 0.03 ^c^	0.42 ± 0.04 ^b^	0.65 ± 0.01 ^a^	0.31 ± 0.01 ^b^

ND: Not detected. SLs-U, Structured lipids enriched with medium-chain fatty acids of 6 h of acidolysis reactions with ultrasonic pretreatment; SLs-N, structured lipids enriched with medium-chain fatty acids of 10 h of acidolysis reactions without ultrasonic pretreatment; Fatty acid composition at sn-1,3 positions was calculated as (3 × total FA-sn-2)/2. ^a–^^c^ Fatty acid in the same location with different letters indicates a significant difference (*p* < 0.05).
